# Ultrasound-Guided Nerve Blocks: Suggested Procedural Guidelines for Emergency Physicians

**DOI:** 10.24908/pocus.v7i2.15233

**Published:** 2022-11-21

**Authors:** Joseph R Brown, Andrew J Goldsmith, Alexis Lapietra, Jose L Zeballos, Kamen V Vlassakov, Alexander B Stone, R Starr Knight, Jennifer Carnell, Arun Nagdev

**Affiliations:** 1 Department of Emergency Medicine, University of Colorado Aurora, CO; 2 Department of Emergency Medicine, Brigham and Women's Hospital Boston, MA; 3 Department of Emergency Medicine, St Joseph's Health Paterson, NJ; 4 Department of Anesthesiology, Perioperative and Pain Medicine, Brigham and Women's Hospital Boston, MA; 5 Department of Emergency Medicine, San Francisco General Hospital San Francisco, CA; 6 Department of Emergency Medicine, Baylor University Houston, TX; 7 Department of Emergency Medicine, Highland Hospital-Alameda Health System Oakland, CA

**Keywords:** Ultrasound Guided Nerve Blocks, Regional Anesthesia, Ultrasound Guided Procedures, Pain Management, Opioids

## Abstract

Acute pain is one of the most frequent, and yet one of the most challenging, complaints physicians encounter in the emergency department (ED). Currently, opioids are one of several pain medications given for acute pain, but given the long-term side effects and potential for abuse, alternative pain regimens are sought. Ultrasound-guided nerve blocks (UGNB) can provide quick and sufficient pain control and therefore can be considered a component of a physician’s multimodal pain plan in the ED. As UGNB are more widely implemented at the point of care, guidelines are needed to assist emergency providers to acquire the skill necessary to incorporate them into their acute pain management.

## Introduction

### Background: Ultrasound-Guided Nerve Blocks: Suggested Procedural Guidelines in The Emergency Department

Acute pain is one of the most frequently encountered complaints in the Emergency Department (ED) 1, 2, 3. Because of the increased awareness of opioid tolerance and potential for downstream abuse, most clinicians have adopted a multimodal pathway for optimal management of acute pain in the ED 4, 5. Ultrasound-guided nerve blocks (UGNB) are a core element of this multifaceted approach to pain 6, 7. Although traditionally used by anesthesiologists for intraoperative anesthesia and the management of post-operative pain, targeted UGNB can also significantly reduce acute pain in ED patients 8, 9, 10, 11, 12, 13, 14, 15, 16, 17, 18. When performed by trained EM physicians, UGNB may also decrease unintended side effects from opioids and procedural sedation, such as hypotension and respiratory depression 19, 20.

Recently, the American College of Emergency Physicians released a Policy Statement on the utilization of UGNB in the Emergency Department, endorsing the use of this procedural skill as a part of multimodal pain control in the ED 21. Further, the American College of Surgeons recently released guidelines on the management of acute pain in trauma patients, endorsing the use of UGNB 19. In the ED, UGNB have been shown to significantly reduce pain, decrease the risk of delirium and decrease patient length of stay without an increase in adverse events 22, 23, 24, 25, 26. 

Ultrasound visualization of both specific nerves and relevant fascial planes greatly enhances clinician comfort in performing UGNB. This allows clinicians to determine a clear needle trajectory while visualizing the needle tip during the entire procedure. This gives ED clinicians the ability to avoid vascular structures and make real-time adjustments that are necessary for effective perineural spread of injectate. Specifically, ultrasound enhances the nerve block duration, decrease complications, and reduce the time spent performing the procedure, therefore providing a more efficacious block 27, 28, 29, 30, 31, 32, 33, 34, 35, 36. 

The following document represents a national collaborative effort by an expert group of physicians to define the scope of practice for UGNB in the ED. We present this document as guidelines for the use of UGNB performed by emergency physicians as a strategy to reduce the reliance of systemic opioids. Specifically, our objectives are as follows:

1. Define and describe the common tasks used when performing a UGNB
2. Describe the core competencies associated with UGNB
3. Suggest a credentialing system for clinicians working in the emergency department
4. Describe the specific complications associated with UGNB.

Due to a lack of published guidelines, we present these guidelines for ED physicians when performing UGNB. 

### Ultrasound-Guided Nerve Block Procedural Department Workflow

#### Consent

A hospital/health system policy should be in place regarding the need for either a verbal or written patient consent before performing UGNB in the ED. Specific attention should be given to LAST syndrome, peripheral nerve injury, procedural pain, bleeding, infection, and block specific complications (i.e. pneumothorax). 

#### Equipment

**Figure 1:**
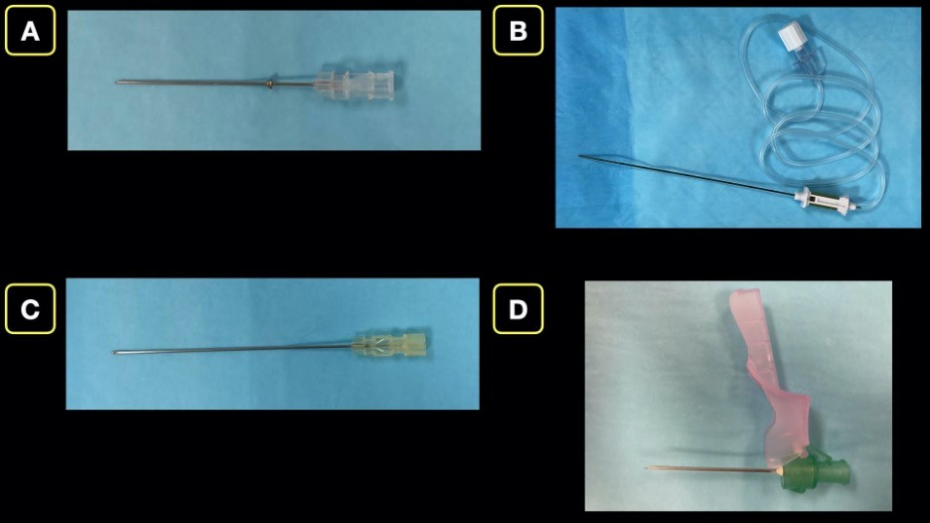
Nerve Block needles. A) Blunt tip 38 mm (1.5 in) Regional Block Needle. B) Blunt tip 100 mm (3.9 in) Regional Block Needle. C) Blunt tip 90 mm (3.5 in) Spinal Needle. D) Sharp tip 40 mm (1.6 in) Cutting Needle.

The operator should choose an ultrasound transducer dependent on the depth of the targeted nerve or fascial plane. UGNB with a required depth less than 5-6 cm can be performed with a high frequency linear probe, while deeper blocks will require a lower frequency curvilinear probe. Echogenic short-beveled needles are recommended if available, otherwise the use of other short-bevel needles, blunt tip spinal needles or Touhy-tip needles are acceptable. Sharp cutting needles are not recommended due to concerns of peripheral nerve injury. (Figure 1). Finally, the needle length should directly be related to the depth of the structures of interest. 

Based on consensus, we recommend continuous ECG, pulse oximetry, and intermittent blood pressure monitoring should be performed on all patients during and after a large volume block (greater than 10 mL) 37. Equipment for airway management and advanced cardiopulmonary resuscitation, as well as lipid emulsion, should be immediately available for managing life-threatening complications.

#### Anesthetic

The anesthetic should be chosen based on the desired length of pain relief. For short relief, 1% lidocaine will provide anesthesia for approximately 45-90 minutes when performing a laceration closure or reduction of joint dislocation. For longer analgesia, ropivacaine or bupivacaine are preferred agents (Table 1). Clinicians should be aware of maximal doses for the anesthetic being used in order to prevent local anesthetic systemic toxicity (LAST) syndrome. Adjuncts can be considered in addition to the local anesthetic to serve as a marker for intravascular injection (epinephrine) and/or further prolong the duration of action (i.e., dexamethasone) 38, 39.

**Table 1:** Anesthetic choices and their respective expected duration of actions for UGNBs in the ED.

**Local Anesthetic**	**Estimated half-life (min)**	**Duration of Anesthesia (h)**	**Maximum Dosage (mg/kg)**	**Common uses**
**1% Lidocaine**	95	1-2	4	Laceration repairs, hand surgery
**0.5% Bupivacaine**	160	6-8	2	Fractures, burns, acute-on-chronic pain indications
**0.5% Ropivacaine**	250	6-8	3	Fractures, burns, post-operative pain, epidural anesthesia

#### Procedure

Prior to starting the procedure, the correct side of the body should be marked and verified by the operator. It is also during this time before the procedure that there should be a pre-procedure briefing. In this the entire team should review the planned procedure and be made aware of potential complications with appropriate interventions, examples include a pneumothorax with corresponding chest tube or LAST and intralipid.

An UGNB is considered a sterile procedure 40. Once sterile, position the ultrasound machine directly in front of the provider and in line with the target of interest. Most UGNBs are performed in-plane with the needle parallel to the direction of the probe with the entire length of the needle (and specifically the needle tip) visualized. In cases where this is not possible, an out-of-plane guided procedure can be performed. In this technique, the needle is advanced perpendicularly to the ultrasound probe and the needle is not visualized in its entirety.

**Figure 2:**
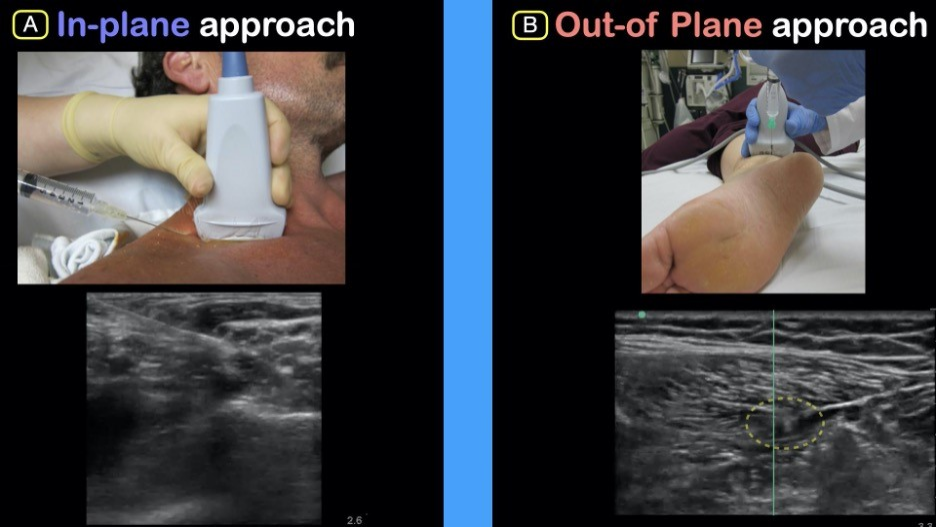
Peripheral Tibial Nerve Block: In-plane approach of needle with visualization of anechoic fluid surrounding nerve on ultrasound.

Once the targeted nerve and/or fascial plane is identified, the needle should be inserted in-plane, if possible. We recommend using small aliquots of sterile saline as the needle tip nears the target nerve or to open the fascial plane (hydrodissection) in order to optimally place anesthetic in the desired location. Using these two aforementioned techniques will help locate the needle tip as well as improve needle tip placement. Once the needle is optimally located gently deposit anesthetic in 1-3 ml aliquots. We recommend aspirating between repeated injections to ensure lack of vascular puncture and clear visualization of anechoic anesthetic on the ultrasound screen (Figure 2).

After completion of the UGNB, the sterile transducer cover should be removed, and low-level disinfection (LLD) performed on the transducer/probe 41. 

#### Monitoring

Currently, there is no evidence-based guidance on the length or need of cardiac monitoring post procedure. However, we find that if greater than 10 cc’s of anesthetic is being used, or if a block is located near large intravascular structures, the patient should be left on the cardiac monitor for a period of observation for the possible development of hypotension or dysrhythmia for at least 60 minutes. This volume of anesthetic assumes using commonly available medications (i.e. Lidocaine, Bupivacaine, or Ropivacaine). Otherwise, we find that 30 minutes is appropriate. During the time of observation, a detailed post-procedure neurologic exam should be documented. If the patient is to be discharged after observation, the patient should be started on a defined multimodal oral pain regimen to avoid the potential of rebound pain when the anesthetic wears off. A clear follow-up system should be present in managing complications such as peripheral nerve injury if they present.

#### Documentation

All UGNBs should be documented in the health record including the clinical indication, a pre-block neurovascular exam, the specific block being performed, the type, concentration and amount of anesthetic, any adjuvants, procedural complications, and a detailed post-block neurologic examination. If the patient is being admitted, a limb alert identifier can be placed to ensure all staff are aware of the motor deficits that may be present post-block. Communication with the ED nursing team and other clinicians caring for the patient is vital to ensure a safe transition of care to the inpatient team. 

### Indications, Training and Credentialing for Point-Of-Care Ultrasound-Guided Nerve Blocks

#### Indications

Single injection ultrasound-guided nerve blocks are ideal for pain control from acute injuries as well as an alternative to procedural sedation for painful procedures. The specific block should be based on the nervous innervation of the affected area, while the choice and volume of anesthetic medication and dose are based on duration of analgesia desired. A complete list of indications affected dermatomes and their associated nerve block can be found in Table 2.

**Table 2:** A current list of UGNBs and their specific indications, affected dermatomes and specific complications. (Con’t on next page).

**Upper Extremity**			
	**Dermatomal Distribution**	**Common Indications**	**Specific Complications**
Superficial Cervical Plexus	Lateral neck extending from clavicle to anteauricular and retroauricular areas	– Clavicular fracture – IJ placement – Earlobe lacerationsSubmandibular abscess and/or Laceration	– Vascular puncture
Interscalene	C5-C7. Complete anesthesia from shoulder to distal humerus. Ulnar nerve is rarely blocked.	– Shoulder dislocation – Proximal humeral fracture – Laceration and/or abscess of upper arm	– Vascular puncture – Intravascular injection – Ipsilateral phrenic nerve palsy – PNIPneumothorax
Supraclavicular	Mid-humerus to distal hand.	– Distal radial fracture – Distal humerus fracture – Deltoid abscess	– Vascular puncture – Intravascular injection – PNIPneumothorax
RAPTIR – Retroclavicular Approach To the Infraclavicular Region, Retroclavicular Block of the Brachial Plexus	Mid-humerus to distal hand	– Arm fractures/dislocations – Large arm abscesses/lacerations	– Vessel puncture – Intravascular injection – PNIPneumothorax
Forearm – Radial, Median, Ulnar	Radial: Radial aspect of dorsum of hand thumb, index finger, middle finger and radial half of ring finger Median: Medial aspect of palmar surface, thumb, index, middle finger and radial aspect of ring finger Ulnar: Ulnar portion of palmar and dorsum of hand and fifth finger. Ulnar aspect of ring finger	– Large lacerations/abscess – Hand fracture/reduction – Finger amputation – Blast injuries	– Vessel puncturePNI
**Trunk**			
	**Regions of Anesthesia**	**Common Indications**	**Specific Complications**
PECS	Axilla, Breast	– Breast abscess – Axillary abscess	
Serratus Anterior	Mid-axilla to anterior T3-9	– Rib Fractures – Thoracic abscess – Tube thoracostomy	– Pneumothorax
Thoracic Erector Spinae (T5-T12)	Truncal nerve INCLUDING posterior aspect. Sympathetic trunk: visceral pain relief	– Posterior rib fracture – Extensive chest wall trauma – Herpes zoster – Acute appendicitis – Vertebral compression fracture	– Pneumothorax
TAP – Transverse Abdominis Plane	T10-12 distribution anterior abdomen	– Appendicitis – Trunk abscess	
Penile	Circumferential penile distribution	– Priapism – Penile trauma	
**Lower Extremity**			
	**Regions of Anesthesia**	**Common Indications**	**Specific Complications**
Transgluteal Sciatic Nerve Block	Posterior thigh to lateral ankle and knee	– Sciatica	– PNI
Fascia Iliaca	Anterior thigh from inguinal ligament to knee	– Hip fracture	– Vessel punctureIntravascular injection
Femoral Nerve	Anterior thigh from greater trochanter to knee	– Femur fracture – Patellar fracture – Thigh abscess	– Vessel puncture – Intravascular injection – PNI
PENG – Pericapsular Nerve Group	Proximal hip joint including medial pelvis and proximal femur	– Acetabular fracture – Pubic rami fracture – Intertrochanteric hip fracture – Femoral neck fracture	– Vessel punctureIntravascular injection
Saphenous Nerve	Medial malleolus of ankle and calf	– Distal tibia fracture	– Intravascular injection
Distal Sciatic Nerve	Distal ⅔ of lower extremity with exception of medial aspect of leg	– Distal tibia/fibula fracture – Achilles tendon rupture – Lateral/Posterior calf abscess/laceration	–Intravascular injection – PNI
Posterior Tibial Nerve	Plantar aspect of foot	– Plantar laceration – Plantar foreign body removal – Plantar abscess – Inadequate anesthesia on extreme medial/lateral aspect of foot	

Training and Credentialing for Point-Of-Care Nerve Blocks 

Since 2001, clear and succinct ultrasonographic credentialing recommendations for POCUS were established by the American College of Emergency Physicians (ACEP). The ultrasonographic guidelines recommended a benchmark minimum of 25-50 quality reviewed scans per modality to demonstrate technical and interpretive ability 42. Conversely for ultrasound guided procedures, 10 quality reviewed scans were recommended. A similar recent guideline for ED transesophageal echocardiography was recently published 43. As UGNBs are an extension of soft tissue and musculoskeletal ultrasound, skill with image acquisition and interpretation will have already been achieved through credentialing in these modalities 44.

The standards presented here may provide a guideline for emergency departments to utilize when credentialing physicians in UGNBs. When looking across other specialties for guidance, currently, the joint committee and the The American Society of Regional Anesthesia and Pain Medicine have omitted any specific recommendations when credentialing providers 45. However, the ACGME has set a standard minimum of 40 peripheral nerve block procedures for anesthesia residents 46. As UGNB education and training has not been standardized within the ED POCUS guidelines, it is important to set a minimum standard. 

Although UGNBs are quite similar to a number of other modalities in the ultrasound guidelines set forth by ACEP, additional required skills are still needed. Operators must be able to visualize and identify specific fascial planes and/or peripheral nerves, visualize the spread of anesthetic with the ability to make adjustments to optimize placement of injectate, detailed understanding of sensory and motor function of specific nerves, and have a thorough understanding of the specific complications associated with UGNBs. A specific skill set needed for proficiency can be found in Table 3.

**Table 3:** Skill Sets associated with Proficiency.

**Image optimization**	**Image Interpretation**	**Needle Insertion and Injection**
Learn the importance of transducer pressures	Identify Nerves	Develop in-plane technique to maximize needle visualization
Learn the importance of transducer alignment	Identify Muscles and Fascia	Develop out-of-plane technique
Learn the importance or transducer alignment	Identify Blood Vessels	Understand the contraindications for each block
Maximizing needle visualization	Identify Bone and Pleura	Visualize correct and incorrect anesthetic placement
	Understand Anisotropy	Minimize unintentional transducer movement
	Identify Acoustic Artifacts	Visualize and recognize intraneuronal needle location
	Identify Needle	

In keeping with ACEP Ultrasound guidelines, providers seeking credentialing in ultrasound-guided nerve blocks should:

1. Complete 2-4 hours of UGNB continuing medical education
2. Complete a minimum of 10 quality reviewed US-guided nerve blocks (of any type) on live patients and simulation models
3. Complete a standardized assessment by a credentialed UGNB provider.

Like transesophageal echocardiography (TEE) and transthoracic echocardiography (TTE), UGNB imaging is a new technical skill. Therefore, a minimum number of UGNB may not be the most accurate method for assessment. A standardized direct observational tool may be more desirable as has been recommended by the recent TEE guidelines. As UGNB is also highly dependent on hand-eye coordination, a standardized direct observational assessment tool is likely more ideal.

As with all new ultrasonographic guidelines, the goal is to offer guidance for optimal performance and safety, and not set unrealistic roadblocks to beneficial novel techniques. These guidelines aim to ensure provider proficiency to provide safe patient care when performing UGNBs, and we based this document on similar anesthesia literature 47, 48. 

### Specific Complications from Ultrasound-Guided Nerve Blocks

#### Peripheral Nerve Injury

Peripheral Nerve Injury (PNI) occurs rarely (less than 0.02%) with 99% of cases resolving within one year 49. If a patient has a motor or sensory deficit, peripheral neuropathy, or poorly managed diabetes we recommend against performing an UGNB given the higher incidence of peripheral nerve injury. To reduce the possibility of PNI, always visualize the needle tip before injecting anesthetic, use a low-pressure injection technique, and a blunt tip needle if available. Additionally, the clinician can ask the patient if there is any discomfort or neuropathic pain during the injection. There should be a clearly defined system for follow-up for patients who persist to have paresthesia, pain or neurological deficits post block. 

#### Local Anesthetic Systemic Toxicity

Local Anesthetic Systemic Toxicity (LAST) is a rare but serious complication that is thought to be due to inadvertent injection of local anesthetic into the vascular system 50, 51. In order to prevent LAST, it is necessary to have clear needle tip visualization throughout the procedure, aspirate frequently prior to, and while, injecting anesthetic, and ensure that the dose of anesthetic being used is under the maximum recommended dose. If your patient exhibits symptoms including vertigo, tinnitus, circumoral numbness, tremors, myoclonic jerks, convulsions, or cardiovascular collapse, the immediate use of intralipid is advised. Intravenous 20% lipophilic emulsion (IVLE) should be easily available to any provider performing UGNBs and a simple protocol readily available for dosing.Specific guidelines in terms of the storage of IVLE and dosing in cases of LAST, should be in place prior to performing this procedure. 

#### Compartment Syndrome

The ability of UGNBs to mask the signs and symptoms of a developing compartment syndrome is debated in the literature 52, 53, 54, 55, 56. Unfortunately, there is no clear evidence to determine which patients could progress to elevated compartment pressures, forcing caution to be used when performing UGNBs in patients with specific injury patterns that are associated with developing compartment syndrome (e.g., high-energy tibia fractures). We recommend discussing with the appropriate consultants prior to performing an UGNB in patients with high-energy mechanism injuries (i.e., tibial shaft fractures, forearm fractures, etc.), or if there is clinical concern for a developing compartment syndrome. For all nerve blocks, a clear interdepartmental/multidisciplinary policy in regard to injury patterns that require consultation before UGNBs will allow for optimal patient selection.

## Conclusion

Acute pain is one of the most frequently encountered complaints in the Emergency Department and it is best addressed through the utilization of a multimodal pathway. Targeted UGNBs should be considered a critical component of a true multimodal approach. UGNBs offer the benefit of minimizing, or avoiding side effects of parenteral medications and procedural sedation. In addition to ACEP’s recent Position Statement on UGNB, these guidelines should assist emergency providers in establishing a protocol for safely incorporating UGNB into the management of their patients with acute pain or prior to performing a procedure.

## Statement of Ethics Approval/Consent

As this piece represents a consensus guideline, patient approval/consent was not obtained.

## Disclosures

Andrew Goldsmith holds a grant from Massachusetts Life Sciences, has received consulting fees from Phillips, Exo, and Bain Capital, and has received honoraria from Indian Health Services. Arun Nagdev holds stock options in Exo Inc., and is a salaried employee of Exo Inc.
